# Negative Mood States Are Related to the Characteristics of Facial Expression Drawing: A Cross-Sectional Study

**DOI:** 10.3389/fpsyg.2020.576683

**Published:** 2020-12-17

**Authors:** Chika Nanayama Tanaka, Hayato Higa, Noriko Ogawa, Minenori Ishido, Tomohiro Nakamura, Masato Nishiwaki

**Affiliations:** ^1^Graduate School of Engineering, Osaka Institute of Technology, Osaka, Japan; ^2^Faculty of Medicine, University of Toyama, Toyama, Japan; ^3^Faculty of Nursing, Setsunan University, Osaka, Japan; ^4^Faculty of Engineering, Osaka Institute of Technology, Osaka, Japan

**Keywords:** convex, enclosure figure, facial expression drawing, feature, length, line, profile of mood states

## Abstract

An assessment of mood or emotion is important in developing mental health measures, and facial expressions are strongly related to mood or emotion. This study thus aimed to examine the relationship between levels of negative mood and characteristics of mouth parts when moods are drawn as facial expressions on a common platform. A cross-sectional study of Japanese college freshmen was conducted, and 1,068 valid responses were analyzed. The questionnaire survey consisted of participants’ characteristics, the Profile of Mood States (POMS), and a sheet of facial expression drawing (FACED), and the sheet was digitized and analyzed using an image-analysis software. Based on the total POMS score as an index of negative mood, the participants were divided into four groups: low (L), normal (N), high (H), and very high (VH). Lengths of drawn lines and between both mouth corners were significantly longer, and circularity and roundness were significantly higher in the L group. With increasing levels of negative mood, significant decreasing trends were observed in these lengths. Convex downward and enclosed figures were significantly predominant in the L group, while convex upward figures were significantly predominant and a tendency toward predominance of no drawn mouths or line figures was found in the H and VH groups. Our results suggest that mood states can be significantly related to the size and figure characteristics of drawn mouths of FACED on a non-verbal common platform. That is, these findings mean that subjects with low negative mood may draw a greater and rounder mouth and figures that may be enclosed and downward convex, while subjects with a high negative mood may not draw the line, or if any, may draw the line shorter and upward convex.

## Introduction

Poor mental health is one of the most important social problems in contemporary society, and countermeasures have been discussed for years (Wagner et al., 2016; McLafferty et al., 2017). Surprisingly, over 8,00,000 people die due to suicide every year, and it is the second leading cause of death among those 15–29 years old in the world (World Health Organization, 2019). The World Health Organization (2019) has thus reported that early identification of individuals with acute emotional distress is of paramount importance to prevent suicide. In addition, many cases of social withdrawal and delinquency are found in developed countries. Such maladaptive states are strongly related to mood changes, such as enhanced anxiety, before falling into these situations (Ellis and Hoskin, 2018). Therefore, further scientific evidence for the simple assessment of anxiety or mood is urgently needed to develop countermeasures against poor mental health.

Many anxiety, mood, or emotion assessment tools have been developed in the past (Watson et al., 1988; Rossi and Pourtois, 2012). These tools are divided broadly into two categories, questionnaire methods and projection methods, and both methods have advantages and disadvantages. Standard questionnaire surveys, such as the Profile of Mood States (POMS), the Positive and Negative affect Schedule (PANAS), the State-Trait Anxiety Inventory (STAI), or the Manifest Anxiety Scale (MAS), are easily available to conduct large-scale investigations (Watson et al., 1988; Rossi and Pourtois, 2012). Because the results generally do not include the subjective views of observers, the reliability and validity of questionnaires are generally high (Elbers et al., 2012). However, the methods have issues of context or order effects, and the obtained answers can include underrated or distorted contents (Shephard, 2003). Although it is difficult to detect underrated and distorted contents in anxiety or mood assessment, the results of questionnaire investigations in other research areas, such as nutrition or physical activity, have been found to differ from actual measured values (Prince et al., 2008; Kaeppler and Erath, 2017). Self-questionnaires are also well-known to increase the intensity and frequency of self-reported symptoms in people with predominant negative affect (Watson and Pennebaker, 1989). Conversely, with projection methods, such as the Baum test and house-tree-human painting, it can be expected that subjects can react freely, and the responses can reflect the complex human nature; they are thus used in a wide range of applications (Stanzani Maserati et al., 2015). However, because the inner aspects of mood and mind are assessed based on drawn size, shape, color, or position, projection methods have no or poor criteria for an objective evaluation, such as specific reference values (Hammer, 1980). Thus, the obtained results may vary depending on the skills of observers, and major issues have to be addressed (Hammer, 1980). Therefore, a mood assessment tool that combines questionnaire and projection approaches has advantages, in that it is simple to administer and convenient to measure and does not require specific skills or expert knowledge compared with other single methods. To the best of our knowledge, no study has attempted to develop such tools.

Several previous studies have examined the relationships between facial expressions and mood or emotion in humans (Darwin, 1872; Ekman, 1970; Matsumoto and Willingham, 2009; Wegrzyn et al., 2017; Magdin et al., 2019; Sato et al., 2019), and mood or emotion changes, such as happiness and anger, modulate facial expressions (Leppanen et al., 2017; Aguado et al., 2019; Scherer et al., 2019). In particular, some facial expressions are universal, reliable markers of discrete emotions when emotions are aroused, and there is no reason to modify or manage the expression (Darwin, 1872; Matsumoto and Willingham, 2009). In addition, the Pain Rating Scale and the Face Pain Scale are widely available for the assessment of levels of pain using facial expression pictures even in children or elderly persons (Hicks et al., 2001; Fadayevatan et al., 2019), but these selective scales can have some issues, such as questionnaire methods. On the other hand, studies using free drawing reported that, when imagining positive topics, the picture that was drawn had a larger size than when imagining negative topics (Burkitt et al., 2003, 2004; Picard and Lebaz, 2010). That is, these findings mean that the effects of mood or emotion can be reflected in the non-verbal methods of free drawing, although standard objective evaluation criteria have not yet been developed. Therefore, if subjects themselves draw a free picture of their mood as a facial expression on a common platform, the facial picture drawn seems likely to indicate their mood by reacting freely and approaching the complex human nature at that time, and standard objective evaluation criteria can be determined or quantified.

Based on this information, we hypothesized that mood states are strongly related to characteristics of facial expression drawing (FACED) on a non-verbal common platform. A study has demonstrated that facial expressions appear easily, especially involving the mouth, and mouth changes corresponding to mood changes have attracted attention in recent years (Wegrzyn et al., 2017; Skiendziel et al., 2019). To obtain the basic data as the first step to the development of new simple mood assessment test, the present cross-sectional study thus aimed to examine whether mood states are related to mouth characteristics of FACED.

## Materials and Methods

### Participants

**FIGURE 1:**
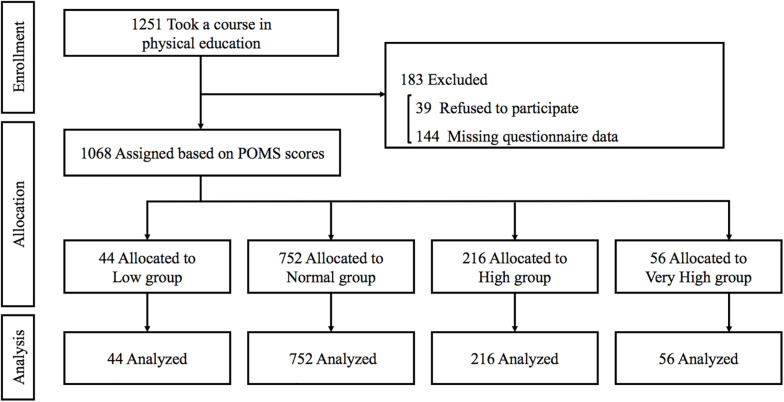
Flow of the study participants.

This cross-sectional observational study involved 1,251 Japanese college students in the Osaka Institute of Technology. Participants were recruited from among the freshmen taking a course in physical education within 1 month from admission. A total of 39 subjects who refused to participate and 144 with missing data were excluded from the statistical analysis. Thus, data were analyzed from 1,068 subjects (male, *n* = 907; female, *n* = 161, Figure 1). The mean age was 18 (range 18–24) years. The purpose, procedures, and risks of the study were explained to each participant. All of them provided their written, informed consent before participating in the study, which was reviewed and approved by the Human Ethics Committee at the Osaka Institute of Technology (approval number: 2016-64) and in accordance with the guidelines of the Declaration of Helsinki.

### Measurements

All investigations were conducted in a quiet air-conditioned room at 22–24°C. Participants’ characteristics such as age, sex, and daily habits were studied first. In addition, the Japanese version of POMS 2nd Edition-Adult Short and FACED were assessed using a questionnaire sheet for exclusive use. Each participant responded to three questionnaires (characteristics, POMS, and FACED) in random order for approximately 10–15 min.

### POMS

In accordance with previous studies (Pribis, 2016; Cho et al., 2018; De Andres-Teran et al., 2019), participants were asked to answer 35 items pertaining to how they felt during the last week, including the present, with responses provided using a five-point scale ranging from 0 (not at all) to 4 (extremely). The questionnaire consisted of seven subscales: Anger-Hostility (AH), Confusion-Bewilderment (CB), Depression-Dejection (DD), Fatigue-Inertia (FI), Tension-Anxiety (TA), Vigor-Activity (VA), and Friendliness (F). Furthermore, the Total Mood Disturbance (TMD) score, as an index of total negative moods, was calculated using the following formula: (AH + CB + DD + FI + TA) − VA. Finally, each TMD score was converted to a standardized TMD score, and this score has been widely used as a psychological indicator (Pribis, 2016; Cho et al., 2018). The reliability of the Japanese version of the POMS was examined in a healthy population, and its validity was examined in people with depression (Yokoyama et al., 1990). The Japanese POMS2 manual indicates that internal consistency (the Cronbach’s alpha) is 0.79–0.96, and in this study, the internal consistency of the measured values was 0.77–0.89.

### FACED and Data Analysis

**FIGURE 2:**
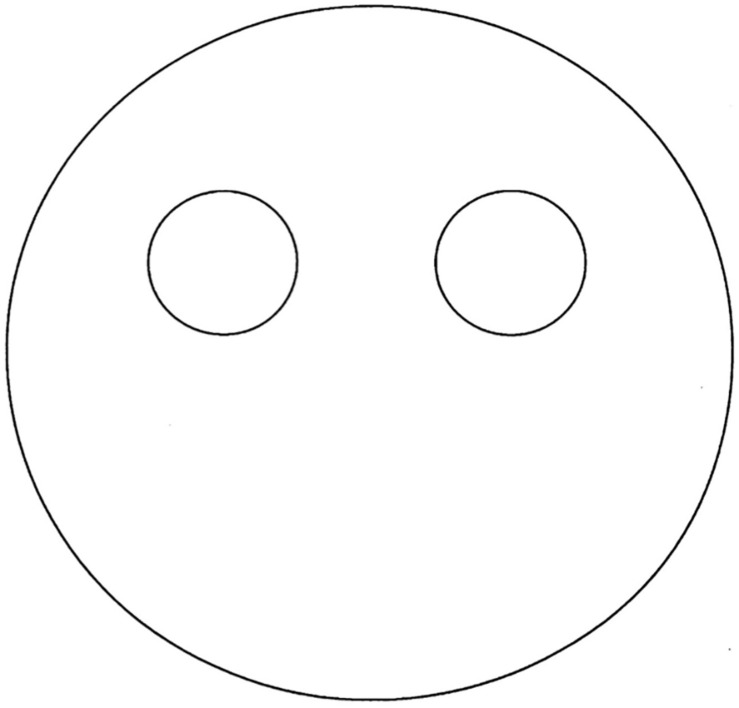
The sheet for facial expression drawing (FACED).

Figure 2 shows the FACED sheet used in the present study. The actual dimensions of the sheet were 14 cm × 14 cm. The sheet consisted of three simple circles: the contours of a face and two eyes. In this survey, participants were asked to express their mood of the past week including the present as a facial expression. They freely drew their mood expression on the FACED sheet with no time or special limitations.

All the drawn FACED sheets were digitized for later off-line manual analysis using an image-analysis software (ImageJ; National Institutes of Health, Bethesda, MD, United States). Each appropriate binarized image was measured more than once, and means were calculated. The lengths of the drawn mouth and between both corners of the mouth were assessed for the size analysis of the drawn mouth. The mouth length was determined by measuring and tracing the drawn mouth line or the circumference of the enclosed figure. Analyses of segmented line tool of the image-analysis software were utilized in the measurements. The length between both corners of the mouth was determined by measuring the direct distance from one corner to the other corner of the drawn mouth. Analyses of the straight-line tool of the image-analysis software were utilized in the measurements. Shape descriptors, such as circularity and roundness of the drawn mouth line or circumference, were also automatically determined. In general, circularity and roundness were calculated using the following formula: 4π × (area)/(circumferential)^2^ and 4 × (area)/[π × (long axis)^2^], respectively. Moreover, based on the above analyzed data, the figure analysis of the drawn mouth was objectively and automatically performed and then categorized by three points: (1) with or without mouths, (2) lines or enclosed figures, and (3) upward convex or downward convex. In particular, upward or downward convex was defined by assessing the position of the vertex toward each baseline, which was determined by the length between both corners. The same single investigator who was unaware of group assignment performed all image analyses. The intraobserver coefficients of variation from the same image analyses on two separate days were 0.9 ± 0.2, 1.3 ± 0.2, 10.5 ± 4.3, and 6.2 ± 4.3% for mouth length, length between both corners, circularity, and roundness, respectively. In addition, the internal consistencies (the Cronbach’s alpha) of length and round shape were 0.75 and 0.95, respectively.

### Statistical Analysis

According to the POMS manual (Heuchert and McNair, 2015) and previous studies (Pribis, 2016; Cho et al., 2018), all participants were assigned to four groups based on standardized POMS TMD scores: low (L), normal (N), high (H), and very high (VH) groups (scores 30 to 39, 40 to 59, 60 to 69, and ≥70, respectively). Continuous data were analyzed by one-way analysis of variance (ANOVA) and analysis of covariance (ANCOVA) that included sex as a covariate. Significant *F*-values were assessed using *post hoc* testing with the Bonferroni correction to identify significant differences among mean values. Differences in non-parametric variables were analyzed by the Kruskal–Wallis test and Scheffe’s method. Trends in continuous data were analyzed by linearity tests and weighted *P*-values were adjusted for sample size. Trends in non-parametric variables were also analyzed by the Jonckheere–Terpstra test. Relationships between parameters were analyzed by Pearson’s correlation, Spearman’s correlation, and stepwise linear multiple regression analyses. All data were statistically analyzed using SPSS Ver. 25 for Windows (IBM Japan Inc., Tokyo, Japan) and Excel Statistics 2016 (SSRI Inc., Tokyo, Japan). Age is presented as mean (minimum–maximum) and data without age are presented as means ± SD. Differences were considered significant at *P* < 0.05.

## Results

### Participants’ Characteristics

Table 1 shows the characteristics of the participants in all groups. No significant differences were observed in age, height, weight, walking during commute time, and living. Significant differences and trends were found in body mass index, sports experience, frequency of eating breakfast, and sleep duration. Table 2 shows the results of the POMS in all groups. The standardized POMS TMD score was higher in the N, H, and VH groups than in the L group, was higher in the H and VH groups than in the N group, and was also notably higher in the VH group than in the H group. A significant increasing trend was observed in the standardized POMS TMD score.

**TABLE 1:** Participants’ characteristics.

Parameters		Total	Low	Normal	High	Very high	*P*-values of one-way ANOVA or Kruskal–Wallis test	Weighted *P*-values of linearity test
Participants, *n* (male)		1,068 (907)	44 (35)	752 (638)	216 (187)	56 (47)	–	–
Age, years		18 (18–24)	18 (18–21)	18 (18–24)	18 (18–22)	18 (18–21)	=0.399	=0.663
Height, cm		169.07.6	169.18.5	169.07.6	168.97.4	168.68.2	=0.980	=0.684
Weight, kg		61.611.1	59.88.9	61.410.5	61.812.1	64.515.5	=0.179	=0.050
Body mass index, kg/m^2^		21.43.2	20.72.6	21.33.0	21.53.3	22.64.7^*†^	=0.021	=0.009
Walking during commute time, min		17.415.7	16.69.3	17.015.2	18.018.1	20.616.9	=0.386	=0.106
Sports experience, years		5.84.0	6.33.9	6.03.9	5.23.8	4.53.9^†^	=0.004	<0.001
Frequency of breakfast, days/week		6.01.8	6.61.1	6.11.7	5.42.2^*†^	5.82.1	<0.001	<0.001
Sleep duration, h		6.01.0	6.01.1	6.11.0	5.91.0	5.61.2^†^	=0.002	=0.001
Living	With family, *n* (%)	904 (84.6)	38 (86.4)	636 (84.6)	179 (82.9)	51 (91.1)	=0.508	=0.922
	Alone, *n* (%)	140 (13.1)	5 (11.4)	100 (13.3)	30 (13.9)	5 (8.9)		
	Others, *n* (%)	24 (2.2)	1 (2.3)	16 (2.1)	7 (3.2)	0 (0.0)		

Age is presented as mean (minimum–maximum) and data without age are presented as means ± SD.*

P < 0.05 vs. low; ^†^*P* < 0.05 vs. normal.

**TABLE 2:** POMS scores by group.

	Total	Low	Normal	High	Very high	*P*-values of one-way ANOVA	Weighted *P*-values of linearity test
Anger-Hostility	3.33.9	0.50.9	2.12.6*	6.14.2^*†^	10.65.3^*†‡^	<0.001	<0.001
Confusion-Bewilderment	7.34.2	1.21.4	6.03.1*	11.12.3^*†^	15.62.5^*†‡^	<0.001	<0.001
Depression-Dejection	5.54.4	0.81.5	3.82.8*	9.93.1^*†^	15.23.3^*†‡^	<0.001	<0.001
Fatigue-Inertia	9.34.4	2.81.9	8.03.4*	13.12.5^*†^	17.21.9^*†‡^	<0.001	<0.001
Tension-Anxiety	10.14.8	3.02.4	8.94.0*	13.83.2^*†^	17.32.0^*†‡^	<0.001	<0.001
Vigor-Activity	9.34.6	15.44.1	9.44.4*	8.04.0^*†^	7.25.2^*†^	<0.001	<0.001
Friendliness	10.43.8	13.64.3	10.43.6*	10.03.6^*†^	9.44.7*	<0.001	<0.001
TMD	26.218.4	−7.23.6	19.310.3*	45.95.9^*†^	68.77.8^*†‡^	<0.001	<0.001
*T* score	53.69.1	37.01.7	50.25.1*	63.32.7^*†^	74.53.9^*†‡^	<0.001	<0.001

*Data are presented as means ± SD.*

POMS, Profile of Mood States; TMD, total mood disturbance.

* *P* < 0.01 vs. low; ^†^*P* < 0.01 vs. normal; ^‡^*P* < 0.01 vs. very high.

### Size Analysis and Shape Descriptors

**FIGURE 3:**
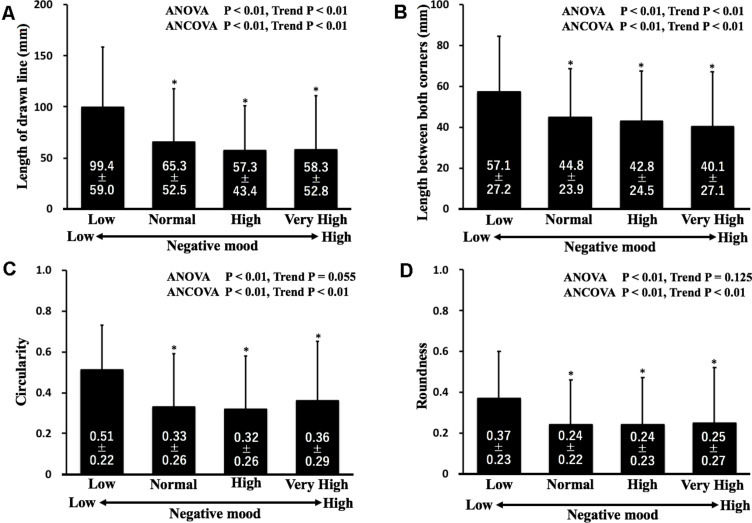
Comparisons of lengths of the drawn line **(A)** and between both corners **(B)**, circularity **(C)**, and roundness **(D)** in each group. ^∗^*P* < 0.01 vs. the low group. Data are expressed as means ± SD.

On ANOVA, the lengths of the drawn mouth and between both corners of the mouth were significantly longer in the L group than in the N, H, and VH groups. Although no significant differences were observed among the N, H, and VH groups, significant decreasing trends were observed in both lengths. In addition, circularity and roundness were significantly higher in the L group than in the N, H, and VH groups. The results remained significant after normalizing the standardized POMS TMD for sex when analyzed by ANCOVA (Figure 3).

### Figure Analysis

**FIGURE 4:**
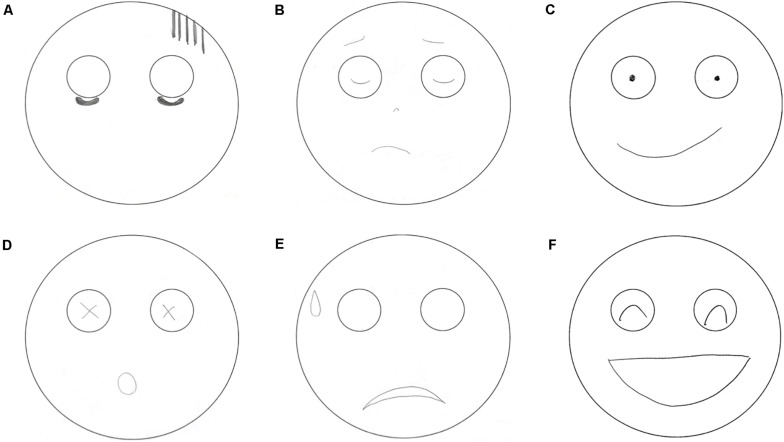
Example drawn mouth figures in facial expression drawing. **(A)** No drawn mouth figure; **(B)** convex upward and line figure; **(C)** convex downward and line figure; **(D)** enclosed figure; **(E)** convex upward and enclosed figure; **(F)** convex downward and enclosed figure.

Figure 4 shows the example results of drawn mouth figures in FACED. A tendency toward a greater number of participants with no drawn mouths was found in the H and VH groups compared with the L and N groups, although no significant difference was observed in with or without drawn mouths. Interestingly, however, especially in the H and VH groups, convex upward was significantly more common, and convex downward was significantly less common than in the L and N groups. Conversely, in the L group, convex upward was significantly less common and convex downward was significantly more common than in the H and VH groups. In addition, a line figure was significantly less common in the L group, and an enclosed figure was significantly more common in the L group than in the other groups (Table 3).

**TABLE 3:** Relationships between drawn figures and negative moods.

Parameters	Low	Normal	High	Very high	*P*-values of Kruskal–Wallis test	*P*-values of Jonckheere–Terpstra test
	(*n* = 44)	(*n* = 752)	(*n* = 216)	(*n* = 56)		
Mouth drawing, *n* (%)	44 (100.0)	731 (97.2)	205 (94.9)	54 (96.4)	=0.222	=0.058
	(*n* = 44)	(*n* = 731)	(*n* = 205)	(*n* = 54)		
Drawn as a line, *n* (%)	21 (47.7)	570(78.0)*	166(81.0)*	46(85.2)*	<0.001	= 0.008
Drawn by enclosed figure, *n* (%)	23 (52.3)	161(22.0)*	39(19.0)*	8(14.8)*	<0.001	<0.001
Drawn convex upward, *n* (%)	0 (0.0)	146(20.0)*	79(38.5)^*†^	24(44.4)^*†^	<0.001	<0.001
Drawn convex downward, *n* (%)	33 (75.0)	317(43.4)*	59(28.8)^*†^	8(14.8)^*†^	<0.001	<0.001

* *P* < 0.01 vs. low; ^†^*P* < 0.01 vs. normal.

### Relationships Between Parameters

The length of the drawn mouth, the length between both corners, and circularity significantly correlated with POMS TMD scores (*r* = 0.163, *P* < 0.001; *r* = 0.142, *P* < 0.001; *r* = −0.088, *P* = 0.005, respectively), but roundness did not (*r* = −0.054, *P* < 0.086). Conversely, convex form (upward or downward convex) and shapes of figure (a line or enclosed figure) significantly correlated with POMS TMD scores (*r*s = −0.081, *P* = 0.008; *r*s = −0.076, *P* = 0.013, respectively), but faces with or without mouth drawing did not (*r*s = −0.050, *P* = 0.101). We thus performed stepwise linear multiple regression analyses of factors affecting POMS TMD scores in three steps. In model 1, the length between both corners, shapes of figure (a line or enclosed figure), and convex form (upward or downward) were entered. In model 2, in addition to the variable in model 1, age, sex, and body mass index were entered. In model 3, in addition to the variable in model 2, walking during commute time, sports experience, frequency of breakfast, sleep duration, and living style were entered. As a result, the length (between both corners) and convex form (upward or downward) were identified as a significant independent factor modulating POMS TMD score in the analysis of full-adjusted mode (Table 4).

**TABLE 4:** Stepwise multiple regression analyses of factors affecting POMS TMD score.

	**Regression coefficient**	**SE**	**β**	***P***	***R*^2^**
Model 1				<0.001	0.042
Constant	39.941	2.194		<0.001	
Convex form	–3.230	0.742	–0.134	<0.001	
Length	–0.104	0.024	–0.133	<0.001	
Circularity	–6.738	2.166	–0.096	=0.002	
Model 2				<0.001	0.042
Constant	39.941	2.194		<0.001	
Convex form	–3.230	0.742	–0.134	<0.001	
Length	–0.104	0.024	–0.133	<0.001	
Circularity	–6.738	2.166	–0.096	=0.002	
Model 3				<0.001	0.080
Constant	41.128	5.365		<0.001	
Length	–0.100	0.026	–0.132	<0.001	
Convex form	–2.858	0.820	–0.120	=0.001	
Sports experience	–0.622	0.189	–0.115	=0.001	
Body mass index	0.543	0.197	0.095	=0.006	
Frequency of breakfast	–0.960	0.354	–0.093	=0.007	

*Model 1: length (between both corners), circularity, shapes of figure (a line or enclosed figure), and convex form (upward or downward).*

*Model 2: model 1 + age, sex, and body mass index.*

*Model 3: full-adjusted model; model 2 + walking during commute time, sports experience, frequency of breakfast, sleep duration, and living style.*

## Discussion

The salient findings of the present study were as follows. Characteristics of the drawn mouth in the L group were significantly longer lengths, higher circularity and roundness, and predominantly enclosed figure and downward convex compared with all the other groups. Conversely, in the H and VH groups, there were tendencies toward shorter lengths and line figures and significantly predominant upward convex compared with all the other groups. To the best of our knowledge, this is the first description of the relationship between states of negative mood and objective characteristics of the drawn mouth part in the FACED and of specific results presented as numerical data.

The POMS is known to be an assessment tool of mood states, and it has been widely used as the gold standard around the world (Pribis, 2016; Cho et al., 2018; De Andres-Teran et al., 2019). Many previous studies demonstrated that this questionnaire has reliability, and its validity is high (McNair et al., 2003). The Japanese version of POMS has also been studied, and it is recognized as a beneficial tool for assessing Japanese individuals (Yokoyama et al., 1990). Furthermore, the present study calculated standardized TMD scores of POMS for each subject based on age and sex (Pribis, 2016; Cho et al., 2018), and participants were divided into four groups according to the scores. Significant differences and trends in TMD scores and each subscale were observed among the groups. Therefore, these results mean that grouping was performed by a highly valid method supported by many previous studies.

In the present study, the drawn FACED sheets were digitized, and appropriate binarized images were analyzed using the image-analysis software. Thus, the data of size and figure obtained from the drawn sheets have high objectivity and validity. In addition, the performed image analysis has high reproducibility. Nevertheless, the present study showed that the drawn mouth in the L group had a significantly longer length and higher circularity and roundness and that an enclosed figure and downward convex were more common in the L group than in all the other groups; that is, subjects with low negative mood drew a larger and rounder mouth and figures that were enclosed and downward convex. Conversely, the characteristics of the H and VH groups were a tendency toward no drawn or a smaller line mouth and upward convex figures predominating, compared with all the other groups; that is, subjects with a high negative mood did not draw or, if any line was drawn, drew the line shorter and upward convex. Stepwise multiple regression analyses also supported the relationships between negative mood states and characteristics of drawn mouths after adjusting the effects of grouping or confounding factors. Therefore, the present findings indicate that the states of negative mood may be related to the size and figure characteristics of drawn mouths on the non-verbal common platform for young adults.

The psychological mechanisms by which negative mood levels were related to the size and figure characteristics of drawn mouths remain unknown. However, these results are considered to come from consciously imaging characteristics of actual facial expressions associated with own mood states, from unconsciously drawing latent mood states, or a combination of the two. From the former perspective, some studies of actual face recognition indicate that when people laugh, the shape of the actual mouth is formed into a downward convex and a rounded outline feature (Lin et al., 2016). According to Darwin, all people, regardless of race or culture, universally express emotions in the face in a similar fashion (Darwin, 1872). Also, pictures or characters of mood, such as emojis, often represent a positive mood as downward roundness convex and a negative mood as a line or upward angular convex (Fadayevatan et al., 2019). Thus, the subjects may image their own mood states and consciously select general facial expression characteristics of positive and negative mood states. From the latter perspective, previous studies indicate that the size of the drawing content altered depending on the states of mood, and positive topics increased the size of drawn pictures (Burkitt et al., 2003, 2004; Picard and Lebaz, 2010). Thus, the experimental task could be actively performed in subjects with low negative mood, and figure characteristics might become larger and longer. In fact, the subjects in the L group showed significantly higher scores on Vigor-Activity and Friendliness as an index of positive mood states. On the other hand, mood states expressed on the faces are relatively easy to hide (Ding et al., 2019), and high negative states are generally known to be hidden by the subjects themselves (Leppanen et al., 2017). In fact, the subjects in the H and VH groups had significantly higher scores of Anger, Hostility, Depression, Dejection, Confusion, Bewilderment, Fatigue, and Inertia states. Thus, they can unconsciously draw the mouth with as little power as possible, and they may not draw the mouth, or if any, they may draw the line shorter and upward convex. However, no direct evidence was obtained in the present study to support this view. Therefore, further studies are needed to elucidate the psychological mechanisms.

Currently, over 8,00,000 people die due to suicide every year, and it is the second leading cause of death among those aged 15–29 years in the world (World Health Organization, 2019). Many cases of social withdrawal and delinquency are also found in developed countries (Ellis and Hoskin, 2018). Thus, to prevent mental health problems, a simple method of quantifying mood states can be of paramount importance. In the present study, although the results were statistically significant, the relationships between negative mood states and the drawn mouth characteristics were not strong. However, since the subjects freely can draw facial expressions on a non-verbal common platform, one possibility is that FACED may partially reflect unconscious latent mood states, which is one of the major advantages of projection methods. In future years, digital image analysis may help to develop objective assessment and criteria, which are elements of questionnaire methods. Thus, FACED might be utilized as a simple non-verbal first screening test for assessment of mood states (i.e., assessing and categorizing low, normal, or high negative mood). Accordingly, further studies of characteristics of other facial parts without mouths, especially the eyes or eyebrow, and determinations of cutoff and reference values are needed to develop a tool for mood assessment.

This study has several limitations. First, the present study involved participants who were only Japanese young male and female college freshmen, and most subjects were male. However, grouping methods were used by age- and sex-standardized scores, and statistical analyses included each group sample size-weighted results and multiple regression analysis. The results of the present study were thus considered unlikely to be strongly affected by the bias of age, sex, and sample size in each group. Second, although each participant responded to POMS and FACED in random order, the results of POMS or FACED might be influenced by the order to answer the questionnaires (i.e., especially in the second questionnaires). Therefore, further experimental studies for examining the reliability or validity of FACED and the effects of order are required to develop a simple mood assessment tool. Finally, the cross-sectional study design limited the ability to determine a cause-and-effect relationship regarding the effects of negative mood states. In terms of a longitudinal design, further studies with a greater range of subjects, such as children or elderly persons, are needed to elucidate whether changes in negative mood states actually affect the size and figure characteristics of drawn mouths.

## Conclusion

The present results suggest that mood states are significantly related to the size and figure characteristics of drawn mouths of the FACED on a non-verbal common platform. That is, the present findings mean that subjects with low negative mood may draw a greater and rounder mouth and figures that may be enclosed and downward convex, while subjects with high negative mood may not draw the mouth, or if any mouth is drawn, may draw a line that may be shorter and upward convex. These specific and actual data therefore could have important implications for the development or improvement of a new simple non-verbal first screening test for assessment of mood states including FACED on a common platform.

## Data Availability Statement

The raw data supporting the conclusions of this article will be made available by the authors, without undue reservation.

## Ethics Statement

The studies involving human participants were reviewed and approved by the Human Ethics Committee at the Osaka Institute of Technology (approval number; 2016-64). The patients/participants provided their written informed consent to participate in this study.

## Author Contributions

CN, HH, and MN conceived and designed the study. CN, MI, TN, and MN performed the study. CN and MN analyzed the data and wrote the manuscript. CN, NO, MI, TN, and MN interpreted the data. All authors approved the final version of the article.

## Conflict of Interest

The authors declare that the research was conducted in the absence of any commercial or financial relationships that could be construed as a potential conflict of interest.

## References

[B1] Aguado L. Parkington K. B. Dieguez-Risco T. Hinojosa J. A. Itier R. J. (2019). Joint modulation of facial expression processing by contextual congruency and task demands. *Brain Sci.* 9:E116. 10.3390/brainsci9050116 PMC6562852 31109022

[B2] Burkitt E. Barrett M. Davis A. (2003). The effect of affective characterizations on the size of children’s drawings. *Br. J. Dev. Psychol.* 21 565–584. 10.1348/026151003322535228

[B3] Burkitt E. Barrett M. Davis A. (2004). The effect of affective characterizations on the use of size and colour in drawings produced by children in the absence of a model. *Educ. Psychol.* 24 315–343. 10.1080/0144341042000211670

[B4] Cho H. B. Bueler C. E. Dimuzio J. Hicks-Little C. Mcglade E. Lyoo I. K. (2018). Negative mood states correlate with laterobasal amygdala in collegiate football players. *Biomed. Res. Int.* 2018:8142631. 10.1155/2018/8142631 PMC5822786 29581986

[B5] Darwin C. (1872). *The Expression of the Emotions in Man and Animals.* London: John Murray.

[B6] De Andres-Teran A. L. Perez-Saez E. Cernuda-Lago A. Sanchez-Vazquez R. (2019). Psychometric properties of Profile of Mood States (POMS) in people with dementia and its application in the evaluation of the effects of therapeutic creative dance. *Rev. Neurol.* 68 190–198. 30805917 10.33588/rn.6805.2018266

[B7] Ding X. Liu J. Kang T. Wang R. Kret M. E. (2019). Automatic change detection of emotional and neutral body expressions: evidence from visual mismatch negativity. *Front. Psychol.* 10:1909. 10.3389/fpsyg.2019.01909 31507485 PMC6716465

[B8] Ekman P. (1970). Universal facial expression of emotion. *Calif. Ment. Health Res. Dig.* 8 151–158.

[B9] Elbers R. G. Rietberg M. B. Van Wegen E. E. Verhoef J. Kramer S. F. Terwee C. B. (2012). Self-report fatigue questionnaires in multiple sclerosis, Parkinson’s disease and stroke: a systematic review of measurement properties. *Qual Life Res.* 21 925–944. 10.1007/s11136-011-0009-2 22012025 PMC3389599

[B10] Ellis L. Hoskin A. (2018). Familial depressive symptoms and delinquency: separate self-reports from mothers and their offspring. *Int. J. Offender. Ther. Comp. Criminol.* 62 1201–1215. 10.1177/0306624x16678939 27864531

[B11] Fadayevatan R. Alizadeh-Khoei M. Hessami-Azar S. T. Sharifi F. Haghi M. Kaboudi B. (2019). Validity and reliability of 11-face Faces Pain Scale in the Iranian elderly community with chronic pain. *Indian J. Palliat. Care* 25 46–51. 30820100 10.4103/IJPC.IJPC_126_18 PMC6388585

[B12] Hammer E. F. (1980). *The Clinical Application of Projective Drawings (Sixth Printing).* St. Springfield: Charles C Thomas Pub Ltd.

[B13] Heuchert J. P. McNair D. M. (2015). *POMS 2 Japanese Manual.* (Yokoyama K. Watanabe K. Trans. in Japanese). Tokyo: Kanekoshobo.

[B14] Hicks C. L. Von Baeyer C. L. Spafford P. A. Van Korlaar I. Goodenough B. (2001). The face pain scale-revised : toward a common metric in pediatric pain measurement. *Pain* 93 173–183. 10.1016/s0304-3959(01)00314-1 11427329

[B15] Kaeppler A. K. Erath S. A. (2017). Linking social anxiety with social competence in early adolescence: physiological and coping moderators. *J. Abnorm. Child Psychol.* 45 371–384. 10.1007/s10802-016-0173-5 27282759

[B16] Leppanen J. Dapelo M. M. Davies H. Lang K. Treasure J. Tchanturia K. (2017). Computerised analysis of facial emotion expression in eating disorders. *PLoS One* 12:e0178972. 10.1371/journal.pone.0178972 28575109 PMC5456367

[B17] Lin Y. Lin H. Lin Q. Zhang J. Zhu P. Lu Y. (2016). A novel three-dimensional smile analysis based on dynamic evaluation of facial curve contour. *Sci. Rep.* 6:22103. 10.1038/srep22103 PMC4766427 26911450

[B18] Magdin M. Benko L. Koprda S. (2019). A case study of facial emotion classification using affdex. *Sensors* 19;E2140. 10.3390/s19092140 PMC6539883 31075816

[B19] Matsumoto D. Willingham B. (2009). Spontaneous facial expressions of emotion of congenitally and noncongenitally blind individuals. *J. Pers. Soc. Psychol.* 96 1–10. 10.1037/a0014037 19210060

[B20] McLafferty M. Lapsley C. R. Ennis E. Armour C. Murphy S. Bunting B. P. (2017). Mental health, behavioural problems and treatment seeking among students commencing university in Northern Ireland. *PLoS One* 12:e0188785. 10.1371/journal.pone.0188785 29236727 PMC5728481

[B21] McNair D. M. Heuchert J. P. Shilony E. (2003). *Research with the Profile of Mood States (POMS) 1964-2002: A Comprehensive Bibliography.* Toronto: Multi-Health Systems.

[B22] Picard D. Lebaz S. (2010). Symbolic use of size and color in freehand drawing of the tree: myth or reality? *J. Pers. Assess.* 92 186–188. 20155568 10.1080/00223890903510464

[B23] Pribis P. (2016). Effects of walnut consumption on mood in young adults-a randomized controlled trial. *Nutrients* 8:E668. 10.3390/nu8110668 PMC5133056 27792133

[B24] Prince S. A. Adamo K. B. Hamel M. E. Hardt J. Connor Gorber S. Tremblay M. (2008). A comparison of direct versus self-report measures for assessing physical activity in adults: a systematic review. *Int. J. Behav. Nutr. Phys. Act.* 5:56. 10.1186/1479-5868-5-56 PMC2588639 18990237

[B25] Rossi V. Pourtois G. (2012). Transient state-dependent fluctuations in anxiety measured using STAI, POMS, PANAS or VAS: a comparative review. *Anxiety Stress Coping* 25 603–645. 21827372 10.1080/10615806.2011.582948

[B26] Sato W. Hyniewska S. Minemoto K. Yoshikawa S. (2019). Facial expressions of basic emotions in Japanese laypeople. *Front. Psychol.* 10:259. 10.3389/fpsyg.2019.00259 30809180 PMC6379788

[B27] Scherer K. R. Ellgring H. Dieckmann A. Unfried M. Mortillaro M. (2019). Dynamic facial expression of emotion and observer inference. *Front. Psychol.* 10:508. 10.3389/fpsyg.2019.00508 30941073 PMC6434775

[B28] Shephard R. J. (2003). Limits to the measurement of habitual physical activity by questionnaires. *Br. J. Sports Med.* 37 197–206. 12782543 10.1136/bjsm.37.3.197 PMC1724653

[B29] Skiendziel T. Rosch A. G. Schultheiss O. C. (2019). Assessing the convergent validity between the automated emotion recognition software noldus facereader 7 and facial action coding system scoring. *PLoS One* 14:e0223905. 10.1371/journal.pone.0223905 31622426 PMC6797095

[B30] Stanzani Maserati M. Matacena C. Sambati L. Oppi F. Poda R. De Matteis M. (2015). The tree-drawing test (Koch’s Baum Test): a useful aid to diagnose cognitive impairment. *Behav. Neurol.* 2015:534681. 10.1155/2015/534681 PMC4484840 26175548

[B31] Wagner S. L. Koehn C. White M. I. Harder H. G. Schultz I. Z. Williams-Whitt K. (2016). Mental health interventions in the workplace and work outcomes: a best-evidence synthesis of systematic reviews. *Int. J. Occup. Environ. Med.* 7 1–14. 26772593 10.15171/ijoem.2016.607 PMC6816521

[B32] Watson D. Clark L. A. Tellegen A. (1988). Development and validation of brief measures of positive and negative affect: the PANAS scales. *J. Pers. Soc. Psychol.* 54 1063–1070. 3397865 10.1037//0022-3514.54.6.1063

[B33] Watson D. Pennebaker J. W. (1989). Health complaints, stress, and distress: exploring the central role of negative affectivity. *Psychol. Rev.* 96 234–254. 2710874 10.1037/0033-295x.96.2.234

[B34] Wegrzyn M. Vogt M. Kireclioglu B. Schneider J. Kissler J. (2017). Mapping the emotional face. how individual face parts contribute to successful emotion recognition. *PLoS One* 12:e0177239. 10.1371/journal.pone.0177239 28493921 PMC5426715

[B35] World Health Organization (2019). *Suicide.* Geneva: World Health Organization.

[B36] Yokoyama K. Araki S. Kawakami N. Tkakeshita T. (1990). Production of the Japanese edition of profile of mood states (POMS): assessment of reliability and validity (in Japanese). *Nihon Koshu Eisei Zasshi* 37 913–918. 2132363

