# Supplementary material for: Negative Mood States Are Related to the Characteristics of Facial Expression Drawing: A Cross-Sectional Study
Source: Front Psychol. 2020 Dec 17;11:576683. doi: 10.3389/fpsyg.2020.576683 (PMC7773925; doi:10.3389/fpsyg.2020.576683)

1．以下の質問にお答えください。

1 現在の居住形態に〇をつけてください	実家　・　一人暮らし　・　その他（　　　　　）
2 通学時間のうち、歩行動作に費やす時間を書いてください	片道1回あたり（　　）分の歩行
3 通学時間のうち、自転車をこぐ時間を書いてください	片道1回あたり（　　）分の自転車
4 過去の運動・スポーツ歴を書いてください	（　　　　　　）を（　　　）年
5 現在のクラブ・サークル所属の有無に〇をつけてください	有　・　無
6 (5で有を選択した方のみ)所属しているクラブ・サークルは運動系ですか	はい　・　いいえ
7 運動・スポーツは好きですか	はい　・　いいえ
8 ここ1週間で朝食をとった頻度を書いてください	週に（　　）回
1 あなたの喫煙習慣に適するものに〇をつけてください	吸っている　・　吸っていない
2 ここ1週間の1日あたりの平均睡眠時間を書いてください	（　　）時間（　　　）分
3 ここ1週間の睡眠の質に適するものに〇をつけてください	非常に良い　・　どちらかというと良い　・　どちらかというと悪い　・　非常に悪い
4 ここ1週間の1日あたりの平均インターネット使用時間を書いてください	学習関連（　　）時間　それ以外（　　）時間
5 あなたの身長・体重を書いてください	身長（　　　）cm　体重（　　　）kg
6 1回30分以上の汗をかく運動を、週2回以上、1年以上続けて行っていますか	はい　・　いいえ
7 日常生活において、歩行または同等の身体活動を1日1時間以上実施していますか	はい　・　いいえ
8 ほぼ同年齢の同性と比較して、歩く速度が速いですか	はい　・　いいえ

2. 今日を含めて過去１週間のあなたの感情状態を、表情として下の顔を用いて表現してください。

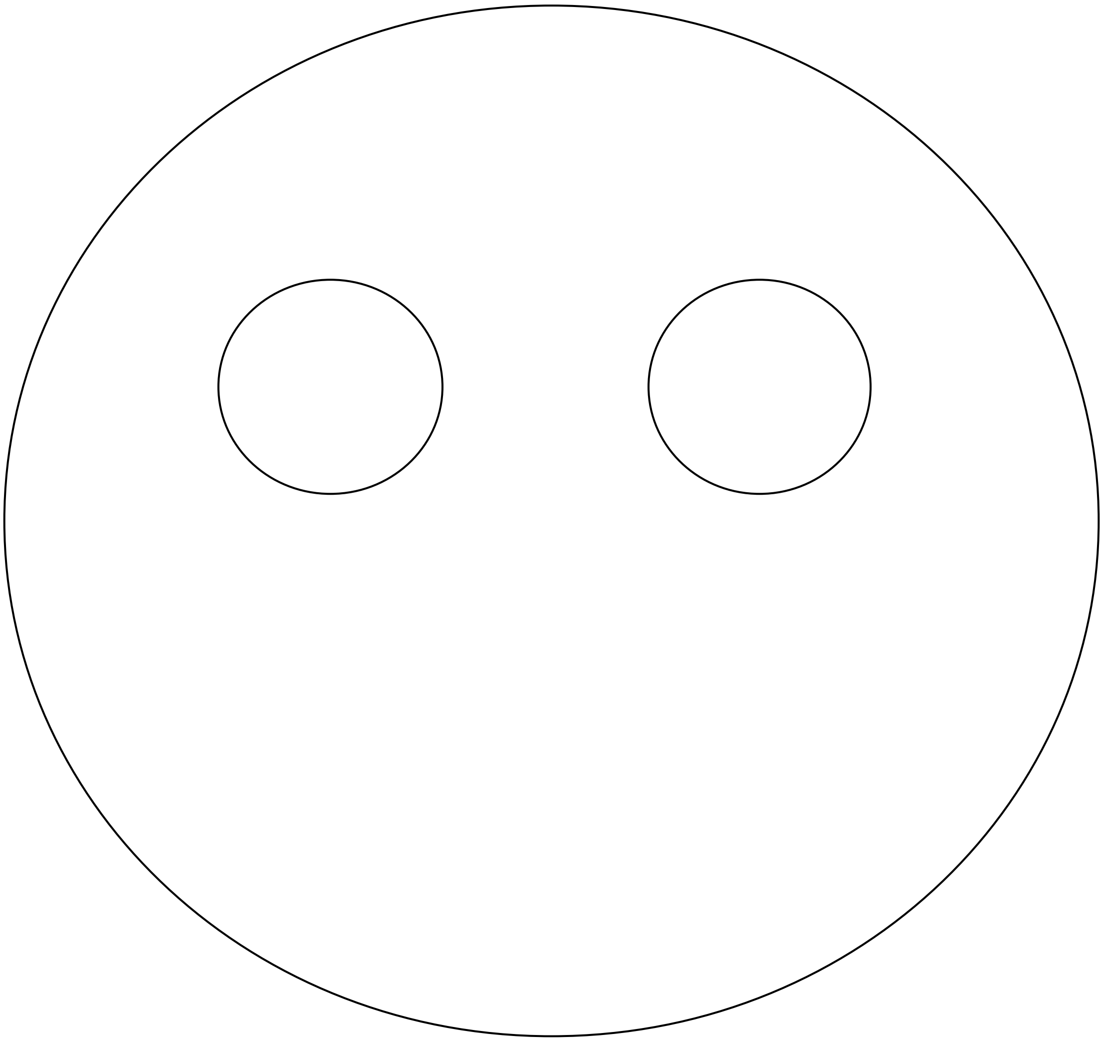

Supplement: Supplementary file 1 [file Data_Sheet_1.PDF]
